# A modified anatomical posterior cruciate ligament reconstruction technique using the posterior septum and posterior capsule as landmarks to position the low tibial tunnel

**DOI:** 10.1186/s12891-024-07176-9

**Published:** 2024-01-18

**Authors:** Yingzhen Niu, Zhen Chen, Lingpeng Jin, Yi Zheng, Zhuangdai Zhang, Fei Wang, Zhenfeng Zhang, Jun Ma, Xiaoqian Men, Jiangtao Dong

**Affiliations:** 1 Department of Joint Surgery, Hebei Medical University Third Hospital, Shijiazhaung, China; 2 Department of Orthopedics, Tiemenguan People’s Hospital, Tiemenguan, China; 3 Department of Hebei Medical University, Shijiazhaung, China; 4 Department of Ultrasound, Hebei Medical University Third Hospital, Shijiazhaung, China

**Keywords:** Posterior cruciate ligament, Tibial tunnel, Posterior septum, Posterior capsule, Anatomy

## Abstract

**Background:**

Lowering the exit position of the tibial tunnel can improve the clinical efficacy of posterior cruciate ligament (PCL) reconstruction, however, there is no unified positioning standard. This study aimed to use novel soft tissue landmarks to create a low tunnel.

**Methods:**

A total of 14 human cadaveric knees and 12 patients with PCL injury were included in this study. Firstly, we observed the anatomical position between the PCL, posterior septum, and other tissue, and evaluated the relationship between the center of the low tibial tunnel (SP tunnel) and posterior septum and distal reflection of posterior capsule, and using computed tomography (CT) to evaluate distance between the center of the SP tunnel with bony landmarks. Then, evaluated the blood vessels content in the posterior septum with HE staining. Finally, observed the posterior septum and distal reflection of the posterior capsule under arthroscopy to explore the clinical feasibility of creating a low tibial tunnel, and assessed the risk of surgery by using ultrasound to detect the distance between the popliteal artery and the posterior edge of tibial plateau bone cortex.

**Results:**

In all 14 cadaveric specimens, the PCL tibial insertions were located completely within the posterior medial compartment of the knee. The distance between the center of the SP tunnel and the the articular surface of tibial plateau was 9.4 ± 0.4 mm. All SP tunnels retained an intact posterior wall, which was 1.6 ± 0.3 mm from the distal reflection of the posterior capsule. The distances between the center of the SP tunnel and the the articular surface of tibial plateau, the champagne glass drop-off were 9.2 ± 0.4 mm (ICC: 0.932, 95%CI 0.806–0.978) and 1.5 ± 0.2 mm (ICC:0.925, 95%CI 0.788–0.975) in CT image. Compared with the posterior capsule, the posterior septum contained more vascular structures. Last, all 12 patients successfully established low tibial tunnels under arthroscopy, and the distance between the posterior edge of tibial plateau bone cortex and the popliteal artery was 7.8 ± 0.3, 9.4 ± 0.4 and 7.4 ± 0.3 mm at 30°, 60° and 90° flexion angels after filling with water and supporting with shaver in posterior-medial compartment of knee joint.

**Conclusions:**

A modified low tibial tunnel could be established in the PCL anatomical footprint by using the posterior septum and posterior capsule as landmarks.

**Supplementary Information:**

The online version contains supplementary material available at 10.1186/s12891-024-07176-9.

## Background

The posterior cruciate ligament (PCL) is indispensable to the radial stability of the knee. Therefore, patients experience various degrees of motor dysfunction after PCL injury [1, 2], and posterior cruciate ligament reconstruction (PCLR) is required to restore physiological function of the knee [3–5]. However, PCLR has a higher rate of failure and revision [6, 7]. Although a number of different factors contribute to PCLR failure, including management of combined injuries and use of different graft types and fixation methods, the tibial tunnel remains a key factor influencing graft stress and clinical outcome [8, 9].

Lower tibial tunnel placement can minimize the effect of “killer turn” and improve graft survival rate in PCLR surgery [10, 11]. However, previous reports have relied mostly on bony landmarks to position the tibial tunnel, and the descriptions were multifarious [12, 13]. Fanelli placed the tibial tunnel in the lateral and inferior region of the PCL tibial insertion, which was below the champagne glass drop-off [8]. Lin et al. positioned the tunnel exit 15 to 18 mm below and slightly lateral to the center of the tibial insertion [11].The bony landmarks varied greatly between patients, resulting in larger variability in low tibial tunnel placement when using these landmarks, which was not conducive to standardization of the clinical technique.

Recent studies have shown that preserving the PCL stump and posterior septum during PCLR is essential for graft survival because these structures are rich in mechanoreceptors and blood vessels [14–16]. Konrads et al. followed up 21 patients with intraoperative preservation of the posterior septum from 6 to 12 months, all patients had no joint effusion, and the mean difference in tibial posterior displacement on stress radiographs was 4.1 mm on the healthy and affected side [17]. Furthermore, the PCL stump can reduce the direct friction between the graft and the tunnel to reduce the “killer turn” [18]. However, many clinicians choose to clean up the PCL stump or use a trans-septum approach to increase the accuracy of tunnel location based on bony landmarks, which undoubtedly increases the PCLR failure rate.

Although previous studies have shown the value of positioning the low tibial tunnel to preserve the posterior septum and stump [19], there are no specific guidelines on how to place the low tibial tunnel without damaging the posterior septum and stump. Therefore, the aim of our study was to explore the appropriate location, and reference landmarks, of the tibial tunnel to retain the PCL stump and avoid damaging the posterior septum. We hypothesized that new soft tissue landmarks will allow surgeons to realize anatomical PCLR and protect the PCL stump and posterior septum while creating the low tibial tunnel.

## Methods

### Source and selection of specimens

A total of 14 freshly frozen unpaired adult knee cadaver specimens (9 males and 5 females) with a mean age of 69.4 ± 5.4 years were dissected in the Anatomy Research Laboratory of Hebei Medical University from 2023.03 to 2023.10, and the study was reviewed by the ethical review committee of Hebei Medical University (No:2022002). The specimens were not treated with formalin, had no history of surgical treatment of the knees, and had intact joint capsules.

### Anatomical observation and low tibial tunnel creation

**Fig. 1:**
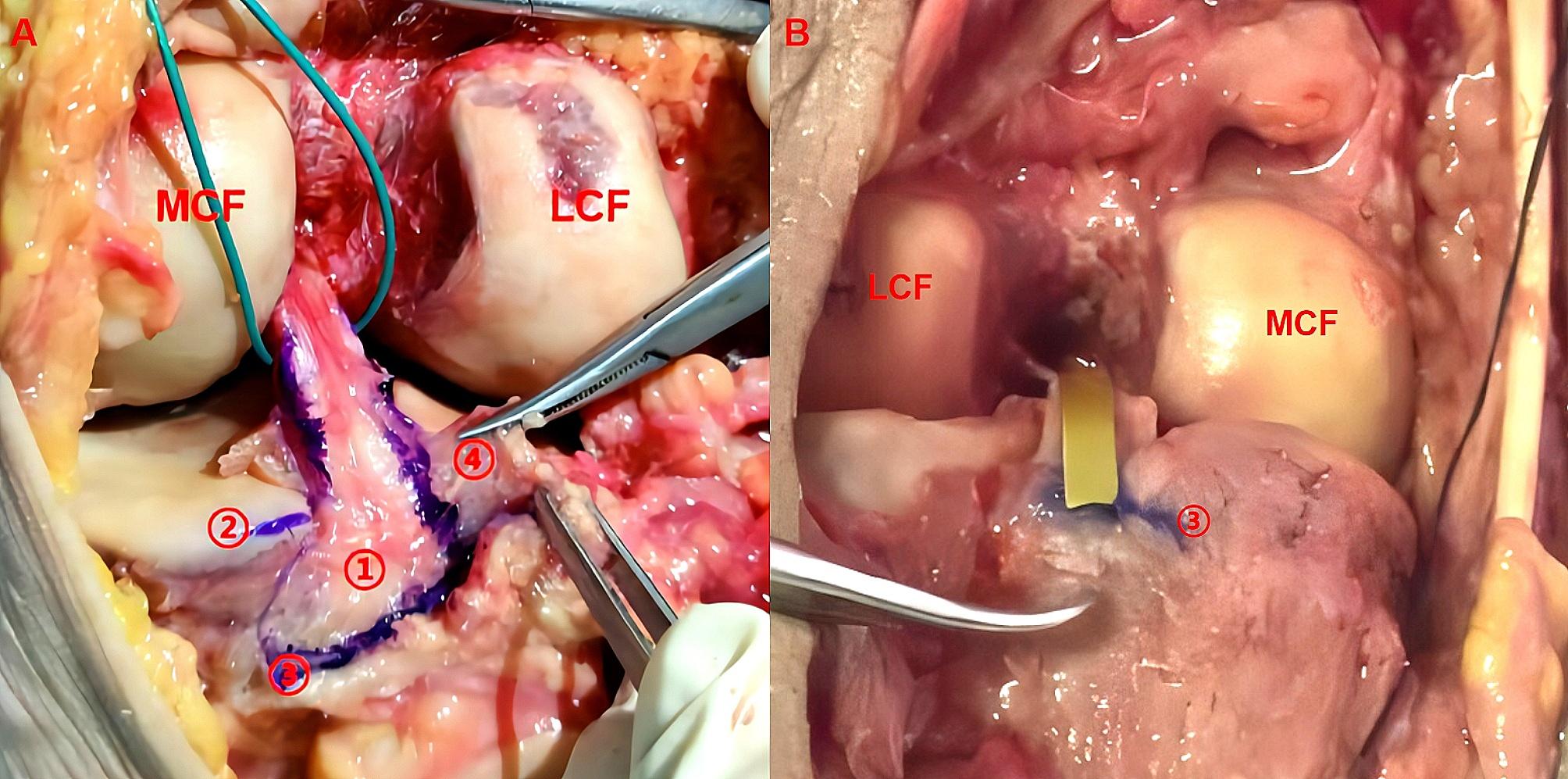
PCL tibial insertion, posterior septum and distal reflection of posterior capsule. **1A**: PCL tibial insertion is located medial to the posterior septum and above the distal reflection of posterior capsule. **1B:** PCL tibial guide locate the center of the tunnel adjusting to posterior septum and the distal reflection of posterior capsule. ① PCL tibial insertion ② lateral tibial plateau ③ distal reflection of posterior capsule ④posterior septum)

All surgical procedures were performed by one senior doctor. The cadavers were fresh frozen at -20 °C and thawed overnight prior to surgery. First, we made an S-shaped incision along the skin and subcutaneous fat layer to expose the popliteal artery and other neurovascular tissues. Second, we cut the medial and lateral heads of the gastrocnemius to expose the capsule, and made an inverted U-shaped incision 8 cm above the femoral condyles [20]. Then, opened the joint capsule and observed the morphology and anatomical relationship between the PCL tibial insertion, posterior septum, and meniscofemoral ligament (MFL) from the medial and lateral views. Subsequently, we placed a PCL tibial guide (Smith & Nephew, USA) along the posterior septum and the distal reflection of the posterior capsule to determine the center of the tibial tunnel (SP tunnel) in the PCL anatomical footprint (Fig. 1). Next, we used 8.0 mm drill to complete SP tunnels and measured the distance from the center of the SP tunnel to the articular surfaces of the tibial plateau and posterior septum. Finally, we observed the integrity of the posterior wall of the SP tunnel and measured the distance between the posterior wall and the distal reflection of the posterior capsule. All reported measurements were performed by the one same postgraduate to reduce inter-observer variability.

### CT reconstruction

**Fig. 2:**
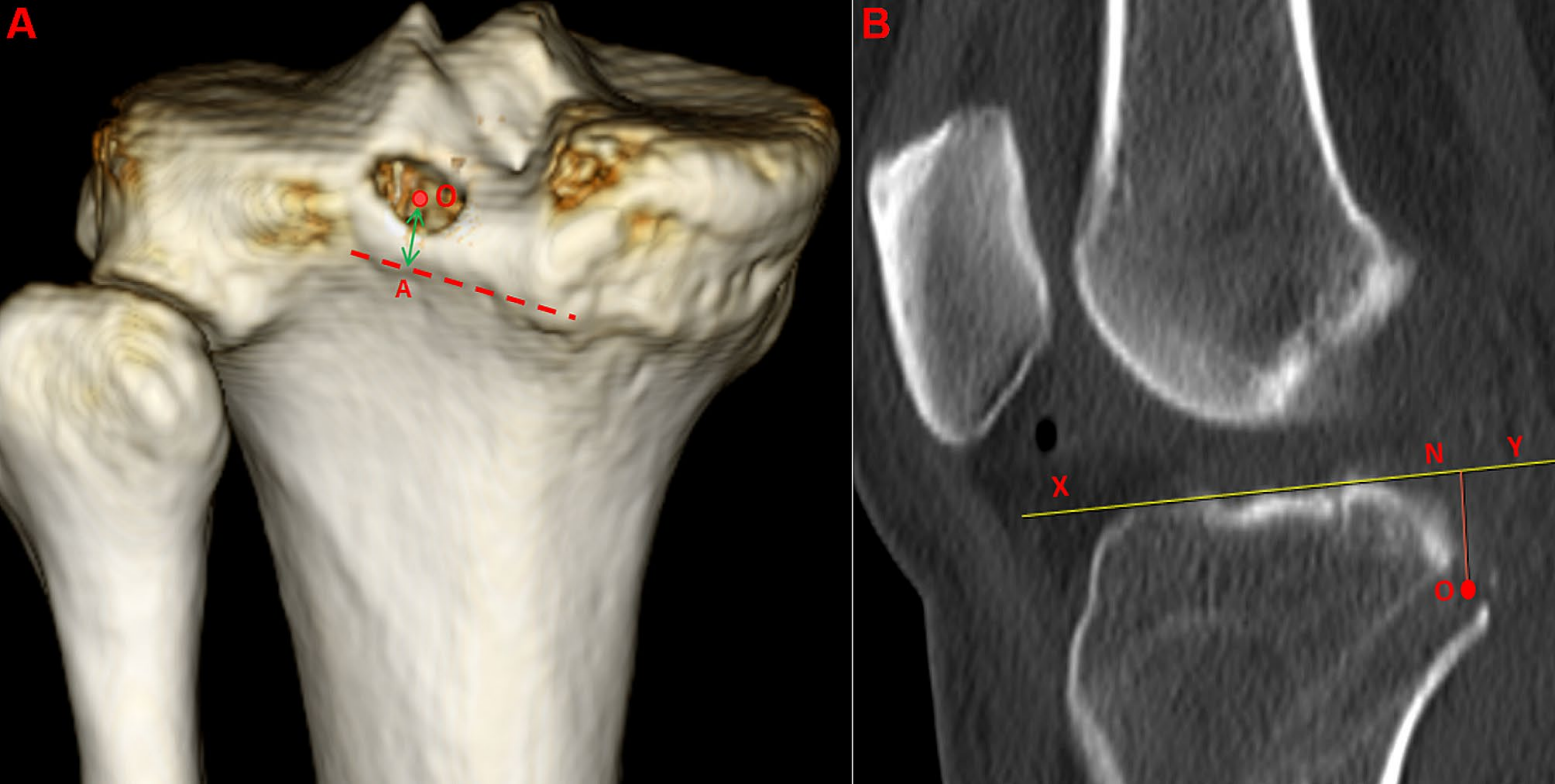
The distance between the center of SP tunnel with bony landmarks. **2A:** The distance between the center of SP tunnel and the champagne glass drop-off; **2B:** The distance between the center of SP tunnel and the articular surface of tibial plateau. O the center of SP tunnel, OA The perpendicular line from the center of SP tunnel to the champagne glass drop-off, XY the articular surface of tibial plateau, ON perpendicular line from the center of SP tunnel to the articular surface of tibial plateau)

All 14 specimens were scanned by CT (Somatom Definition AS 128, Siemens, Germany) after the SP tunnel was established by using Mango (Medical software). The position of the tunnels were evaluated by measuring the center of SP tunnel from the champagne glass drop-off on CT three-dimensional reconstruction model (Fig. 2A), and the distance between the center of SP tunnel and the the articular surface of tibial plateau on CT sagittal image (Fig. 2B). The data were assessed for reliability by two private experienced physicians. Moreover, the reliability of the 14 SP tunnels were assessed again after two months by one physician.

### Histological analysis

All posterior septum and posterior capsule specimens obtained from the dissection were unfolded as far as possible. After dehydration, transparency, paraffin embedding and fixation, it was sliced with a thickness of 5 μm. Then, Hematoxylin-eosin (HE) staining was performed to observe the extent and main distribution area of blood vessels in the anterior, middle and posterior regions of the posterior septum and posterior capsule under 4 × 10 and 10 × 10 optical microscopes.

### Arthroscopic localization and ultrasound measurement

**Fig. 3:**
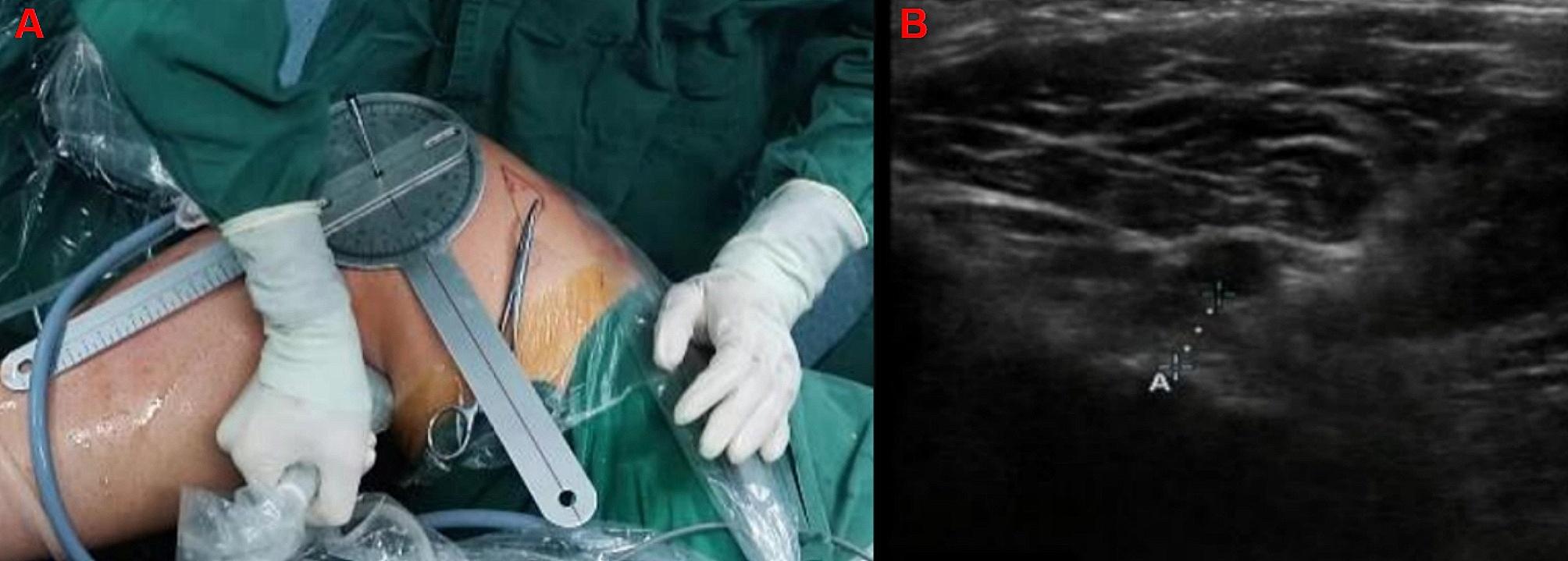
Ultrasound measures the distance between the posterior edge of the tibial plateau bone cortex and the popliteal artery at different flexion angles of the knee. **3A:** Measure the distance between at knee flexion 90; **3B:** Ultrasound measures the distance between the posterior edge of the tibial plateau bone cortex and the popliteal artery)

A total of 12 patients (8 males and 4 females; 38.5 ± 18.2 years) were selected for PCLR between July 2022 and May 2023. Before operation, we measured the distance between the posterior edge of tibial plateau bone cortex and the popliteal artery by ultrasonic (Acuson Sequoia 512 ultrasonic instrument, 15L8W broadband linear array probe, frequency 8.0-13.0 MHz) under knee flexion of 30°, 60°and 90°respectively after the patient was successfully anesthetized (Fig. 3A). Then, after exploring the posterior compartment of the knee joint with arthroscopy, we injected water into the knee joint and checked the distance the posterior edge of tibial plateau bone cortex and the popliteal artery with ultrasound (Fig. 3B). Next, we supported the posterior capsule with a shaver to repeat the distance test under ultrasound again, and observed the anatomical relationship between the PCL, posterior septum and posterior capsule under arthroscopy. During the operation, 3000 ml of normal saline was used with continuous constant pressure, and the saline was set as 80 cm height beyond the operating table to maintain the constant amount of perfusion pressure. Finally, we used a PCL tibial guide (Smith & Nephew, USA) to locate the tunnel exit adjacent to the posterior septum and the distal reflection of the posterior capsule. All arthroscopic surgeries were performed by one senior doctor.

### Statistical analysis

Statistical analysis was performed using SPSS 22.0 software. Count data were expressed as X, t-test was used for normally distributed continuous variables, Rank test was used for non-normal data, and ρ < 0.05 was considered statistically significant. The Interclass Correlation Coefficient (ICC) was used to evaluate the reliability of CT measurements within and between observers; ICC greater than 0.9 indicated excellent reliability.

## Results

### PCL tibial insertion and surrounding soft tissues

**Fig. 4:**
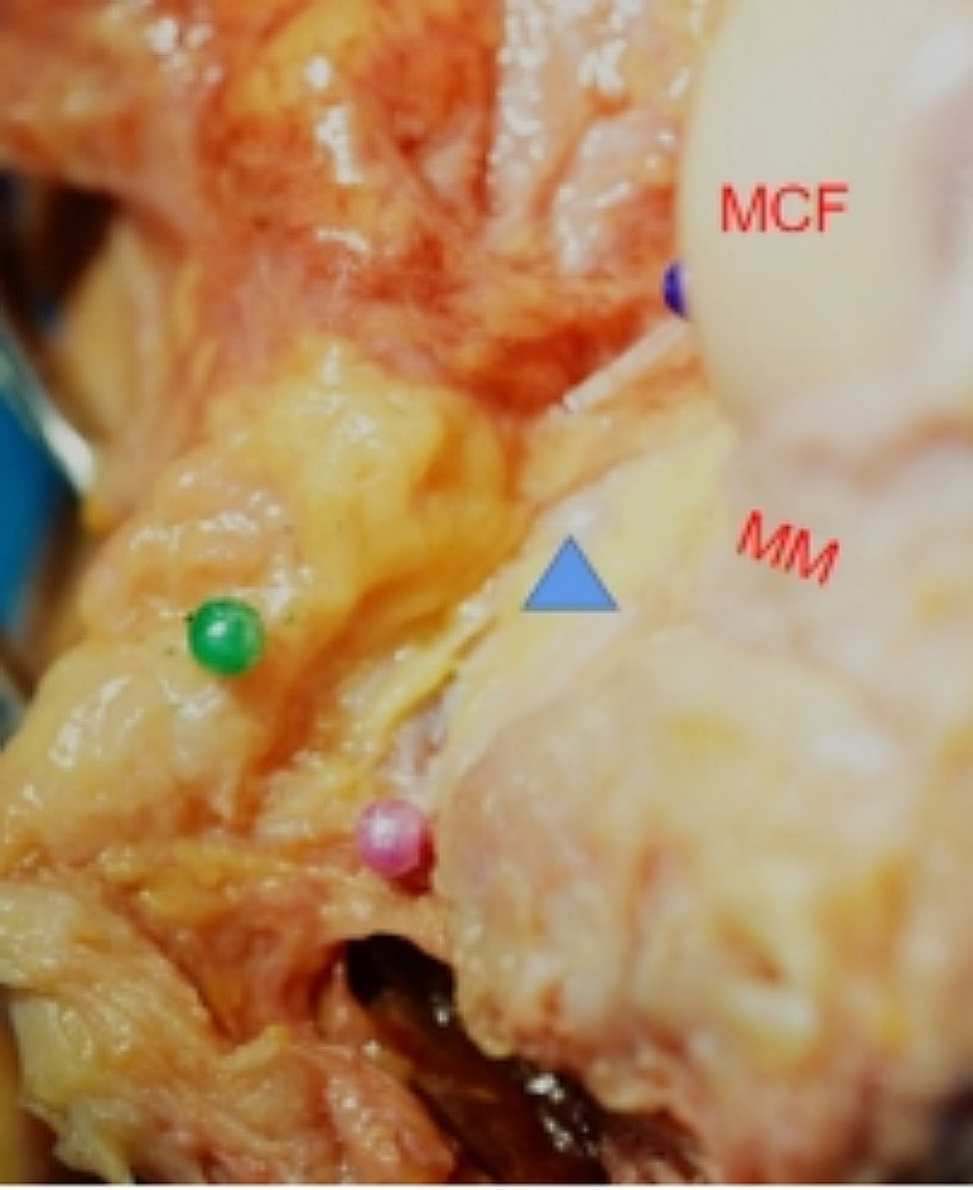
Posterior capsule reflection. The distal reflection of the posterior capsule was closely connected to the PCL tibial insertion and just above the champagne glass drop-off. Pink ball = capsule reflexion, green ball = posterior septum, MCF = medial femoral condyle, MM = medial meniscus, △= PCL)

In all 14 specimens, the PCL tibial insertions were located medial to the posterior septum and could not be observed in the posterolateral compartment (Fig. 1). The shape of the PCL tibial insertion was similar to a right-angled trapezoid with an area of approximately 153.5 ± 11.0 mm² (*p* > 0.05). The superior and inferior boundaries of PCL tibial insertion were adjacent to the posterior root of the medial meniscus and the distal reflection of the posterior capsule, approximately 9.1 ± 1.0 and 14.4 ± 0.6 mm respectively. The medial boundary was located just posterior to the medial tibial intercondylar crest and the lateral boundary was close to the posterior septum, approximately 15.4 ± 0.9 and 13.0 ± 0.4 mm respectively. In addition, we found there were no obvious distance between the distal reflection of the posterior capsule and the champagne glass drop-off (Fig. 4).

### The SP tunnel and posterior septum

All SP tunnels were successfully established and all SP tunnels retained an intact posterior wall. The distances between the center of SP tunnel and articular surface and posterior septum were 9.4 ± 0.4 mm 4.5 ± 0.3 mm, respectively. The distance between the inferior edge of SP tunnel and distal reflection of posterior capsule was 1.6 ± 0.3 mm. In the CT sagittal image and three-dimensional reconstruction model, the distances between the center of the SP tunnel and the the articular surface of tibial plateau, the champagne glass drop-off were 9.2 ± 0.4 and 1.5 ± 0.2 mm, and the ICC values indicating inter-observer reliability (Table 1) and intra-observer reliability (Table 2) for the SP tunnel location related to the PCL tibial insertion in CT.

**Table 1:** The ICC (95%CI) of the SP tunnel location to PCL tibial insertion between two physicians

Physician	Center of SP tunnel to the articular surface of tibial plateau (mm)	Inferior edge of SP tunnel to the champagne glass drop-off (mm)
1	9.186 ± 0.374	1.543 ± 0.241
2	9.164 ± 0.326	1.564 ± 0.238
ICC	0.932	0.925
95%CI	0.806–0.978	0.788–0.975
P	<0.05	<0.05

**Table 2:** The ICC (95%CI) of the SP tunnel location to PCL tibial insertion within one physician

Physician 1	Center of SP tunnel to the articular surface of tibial plateau (mm)	Inferior edge of SP tunnel to the champagne glass drop-off (mm)
First measurement	9.186 ± 0.374	1.543 ± 0.241
Second measurement	9.221 ± 0.378	1.536 ± 0.235
ICC	0.946	0.959
95%CI	0.846–0.982	0.878–0.987
P	<0.05	<0.05

### Histological study

**Fig. 5:**
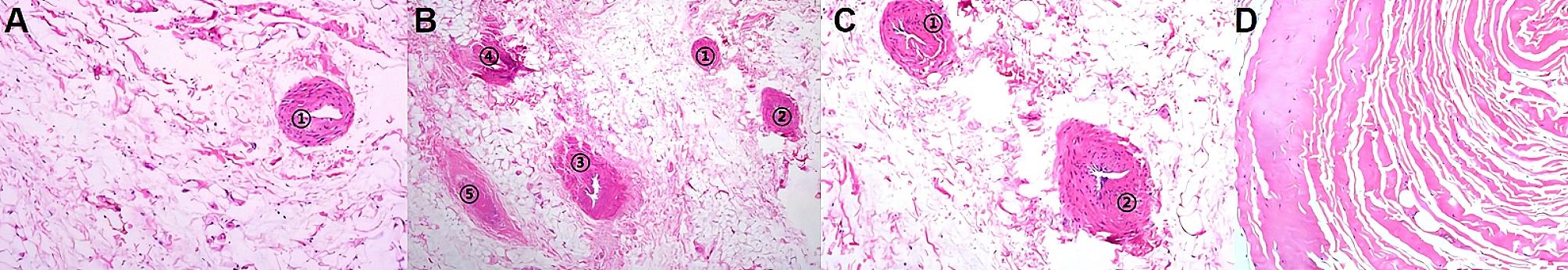
HE staining. **5A:** The vascular tissue in posterior capsule under 4 × 10 microscope; **5B:** The bottom of the posterior septum is rich in vascular tissue under 4 × 10 microscope; **5C:** The vascular tissue in septum under 10 × 10 microscope; **5D:** There are no vascular tissue under 10 × 10 microscope. ①②③④⑤: vascular structure)

It had less continuous vascular tissue could be observed in the middle region of the section, and little or no vascular tissue could be seen in the surrounding region of the section in the posterior capsule section (Fig. 5A), while a large amount of vascular tissue was distributed within the posterior septum sections under a 4 × 10 magnification microscope. It is noteworthy that the vascular tissue in the bottom of the posterior septum had more vascular distribution compared with the posterior capsule (Fig. 5B). The vascular structures in the posterior septum were clearly visible and distributed in a continuous pattern (Fig. 5C), while only a large number of collagen structures with a small amount of vascular tissue were visible in the capsule tissue section under a 10 × 10 magnification microscope (Fig. 5D).

### Arthroscopic localization and measurement

**Fig. 6:**
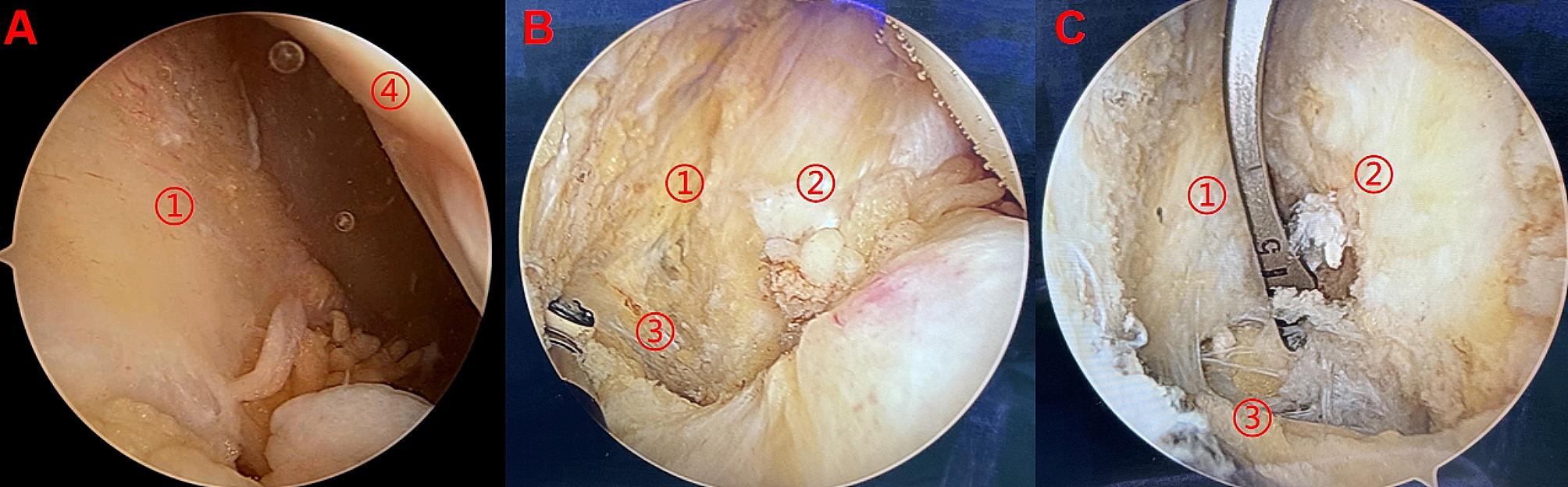
Observation of PCL and surrounding soft tissues under arthroscopy. **6A:** The posterior septum as a curtain in the posterolateral compartment; **6B:** PCL stump, posterior septum and distal reflection of posterior capsule; **6C:** PCL tibial guide locate the center of the tunnel adjusting to posterior septum and the distal reflection of posterior capsule. ①posterior septum ②PCL stump ③distal reflection of posterior capsule ④Lateral femoral condyle)

Under arthroscopic, we can only observe the posterior septum as a curtain in the posterolateral compartment without PCL (Fig. 6A), but it can be clearly observed that the shining white fibers of PCL were located medial to posterior septum after arthroscopy entered the posterior medial chamber (Fig. 6B). The posterior capsule was supported posteriorly with a shaver to identify the junction zone of the posterior septum and the distal reflection of the posterior capsule. Finally, the PCL tibial guides (STAR, CHINA) were placed on the junction zone (Fig. 6C). All patients underwent preoperative and intraoperative ultrasound examination, and the distances between the posterior edge of tibial plateau bone cortex and the popliteal artery in patient at three different flexion angles were shown in Table 3.

**Table 3:** The distance between the posterior edge of tibial plateau bone cortex and popliteal artery at different angles of knee flexion and in different States

State	Knee flexion			P^1^	P^2^	P^3^
	30° (mm)	60° (mm)	90° (mm)			
No water injection	6.5 ± 0.3	6.9 ± 0.2	6.0 ± 0.4	<0.01	<0.01	<0.01
Simple intra-articular injection with water	7.9 ± 0.3	7.6 ± 0.2	7.6 ± 03	<0.01	<0.05	>0.05
Intra-articular injection with water and assist distraction with shaver	7.8 ± 0.3	9.4 ± 0.4	7.4 ± 0.3	<0.01	<0.01	<0.01
P^4^	<0.01	<0.01	<0.01	-	-	-
P^5^	<0.01	<0.01	<0.01	-	-	-
P^6^	>0.05	<0.01	>0.05	-	-	-

P^1^*P* value between the knee flexion 30° and 60° ; P^2^*P* value between the knee flexion 30° and 90°; P^3^*P* value between the knee flexion 60° and 90°; P^4^*P* value between the No water injection and Simple intra-articular injection with water; P^5^*P* value between the No water injection and intra-articular injection with water and assist distraction with shaver; P^6^*P* value between the Simple intra-articular injection with water and Intra-articular injection with water and assist distraction with shaver

## Discussion

The key finding of this study was that we could establish a safely and highly reproducible low tibial tunnel within the PCL tibial insertion by using the posterior septum and the distal reflection of the posterior capsule as soft tissue landmarks.

Previous studies have found that the posterior septum not only distributes the middle genicular artery vascular network, but also contains a large number of type II and type IV mechanoreceptors [14, 17]. However, few studies have assessed the relationship between the PCL and the posterior septum and the results were controversial [15, 21]. In our study, we found that the PCL tibial insertions were located entirely medial to the posterior septum and there were no obvious distance between the distal reflection of the posterior capsule and the inferior boundary of the PCL tibial insertion, so we regarded the posterior septum and distal reflection of the posterior capsule as the lateral and inferior boundary of the PCL tibial insertion. Thue, we chose them as soft landmarks to build the SP tunnel. We found the center of the SP tunnel was 9.4 ± 0.4 mm from the articular surface of the tibial plateau, and the distance between the center of the SP tunnel and the articular surface of tibial plateau was 9.2 ± 0.4 mm on CT three-dimensional reconstruction model. These data are similar to the results reported by Fanelli and Lin [8, 11], indicated the posterior septum and capsule could be used as other bony landmarks to effectively avoid the graft rubbing and impinging on the medial condyle wall [22]. In addition, we found that the distance between the posterior wall of the SP tunnel and the distal reflection of the posterior capsule was 1.6 ± 0.3 mm and all 14 SP tunnels were not burst. This means that using the distal reflection of the posterior capsule as a landmark, we could achieve the lowest position of the tunnel to achieve anatomical reconstruction, while maintaining the integrity of the posterior wall and retaining the PCL stump as much as possible.

Previous mechanical studies confirmed that low tibial tunnels can reduce stress on the graft and improve the survival rate [23]. Tang et al. found that the graft was subjected to the lowest peak stresses when placed 5–20 mm inferior and 5–10 mm lateral to the anatomical insertion of the PCL [24]. This region included the non-anatomical tunnel below the champagne glass drop-off proposed by Fanelli [8]. However, other studies demonstrated that graft stability would be improved when placed closer to the anatomic footprint of the ligament [25, 26]. The SP tunnel proposed in our study, which was just above the champagne glass drop-off, is within the low-tension zone, meaning the SP tunnel can realize an anatomical reconstruction, with a good mechanical results.

For many years, we have used the double posterior medial approach during PCLR to protect the posterior septum, with the knee in the “4” position and flexed at 60 degrees, and no complications were found during the hospitalization. When positioning and creating the tunnel, the shaver can play the role of planing, attracting and spreading at the same time during the operation, so that the neurovascular tissues were drawn toward the posterior part of the operating field. Taking the longitudinal axis of the posterior septum as the reference, the shaver was directed dorsally toward the posterior capsule, and a part of synovial tissue was cleared between the posterior capsule and the PCL stump, with the space being supported and cleaned until the distal reflection of the posterior capsule was revealed [27]. In addition, Yoo et al. measured the position of the general tibial tunnel was1cm below the tibial plateau by MRI axial position, and the average distance from the popliteal artery was 2.7 and 9.2 mm when the knee joint was straight position and flexed 90° [28]. The data obtained by preoperative and postoperative ultrasound in this study show that the distance between popliteal artery and the posterior edge of tibial plateau is larger with the increase of knee flexion angle under the condition of double posterior medial approach, and the distance reached the maximum when the knee joint flexes 60°. However, the tibial tunnel established on the medial boundary of the tibial anatomical insertion can be avoided from the lateral nerve and blood vessels, which has better safety.

There were some limitations in this study. First, only 14 specimens were used in the anatomical experiments and only 12 patients with PCL injury were observed under arthroscopy. However, despite these small cohort sizes, the combination of anatomy and clinical practice can improve the reliability of the research results. Second, the risk of the tunnel wall being compromised increases closer to the champagne glass drop-off. However, the SP tunnels in this study all retained an intact posterior wall according to the distance between the posterior edge of SP tunnel and the champagne glass drop-off was 1.6 ± 0.3 mm. Finally, although preserving the posterior septum showed anatomical and mechanical advantages, we still need long-term comprehensive follow-up of clinical cases.

## Conclusions

The PCL tibial insertion was located medial to the posterior septum, with the posterior septum and distal reflection of the posterior capsule as lateral and inferior boundaries. A modified low tibial tunnel could be established in the PCL anatomical footprint by using the posterior septum and posterior capsule as combined soft tissue landmarks.

### Electronic supplementary material

Below is the link to the electronic supplementary material.

**Supplementary Material 1**: STROBE Statement—checklist of items that should be included in reports of observational studies

## Data Availability

The data analyzed in this study were taken from anatomy study, which can be found from Pubmed. The original data can be contacted with the correspondent: Jiangtao Dong, Email: djtloveyz@outlook.com, Tel: 0311-88602801.
